# The Early Youth Engagement (EYE‐2) Intervention in First Episode Psychosis Services: Definition and Implementation of a Therapeutic Motivational–Engagement Model for Early Intervention in Psychosis Key Workers

**DOI:** 10.1111/eip.70270

**Published:** 2026-10-16

**Authors:** Kathryn Greenwood, Richard O. de Visser, Victoria Smallman, Claire Vella, Stuart Clark, Rebecca Webb, Anastacia O'Donnell, Louise Johns, Philippa Garety, Emmanuelle Peters, Shanaya Rathod, Peter Phiri, Sunil Nandha, Belinda Lennox, Paul French, Jo Hodgekins, David Fowler, Heather Law, Andrew Healey, Stephen Bremner, Rose Thompson, Carl May

**Affiliations:** ^1^ School of Psychology University of Sussex Falmer UK; ^2^ Research and Development Department Sussex Partnership NHS Foundation Trust Hove UK; ^3^ Department of Primary Care and Public Health Brighton and Sussex Medical School Falmer UK; ^4^ Department of Psychiatry University of Oxford Oxford UK; ^5^ Institute of Psychiatry Psychology and Neuroscience King's College London London UK; ^6^ South London and Maudsley NHS Foundation Trust London UK; ^7^ Research and Development Department Southern Health NHS Foundation Trust Southampton UK; ^8^ Pennine Care NHS Foundation Trust Ashton‐Under‐Lyne UK; ^9^ Manchester Metropolitan University Manchester UK; ^10^ University of East Anglia Norwich UK; ^11^ Greater Manchester Mental Health NHS Foundation Trust Greater Manchester UK; ^12^ McPin Foundation London UK; ^13^ Faculty of Public Health and Policy London School of Hygiene and Tropical Medicine London UK

**Keywords:** early intervention, engagement, key worker, model, psychosis

## Abstract

**Background:**

Psychosis is associated with high disability and societal costs. Early Intervention in Psychosis (EIP) services enhance mental health outcomes, but 15% disengage and more engage intermittently. There is no EIP‐specific therapeutic model for how Key Workers should maximize engagement.

**Aims:**

To identify factors that define the Early Youth Engagement (EYE) therapeutic model for Key Workers in EIP services and affect implementation.

**Methods:**

The design was a cross‐sectional qualitative thematic analysis and the final phase of an iterative mixed‐methods model development. Participants were 15 EIP clinicians who took part in the EYE pilot project and were interviewed regarding core therapeutic ingredients and implementation.

**Results:**

The final EYE‐2 therapeutic model was defined by 10 sub‐themes forming three therapeutic process themes (Psychoeducation; The Motivational–Engagement relationship; An Early Systemic Approach) and an outcome theme (Achieving Meaningful Recovery Outcomes). Fourteen implementation‐related subthemes were mapped onto Normalization Process Theory constructs. They revolved largely but not exclusively around the more tangible psychoeducation component.

**Conclusions:**

The study defines, for the first time, the key components of a Therapeutic Motivational–Engagement Model for Key Workers in EIP services derived from service user and carer perspectives on needs. The clarity regarding core therapeutic processes to better support engagement enables the potential to standardize and reduce complexity in the Key Worker role. This clarity is important because key workers' role confidence impacts on therapeutic relationships and outcomes. The model is well‐liked, easy to implement and supported by psychoeducational resources adapted for people from minority ethnic populations. Effectiveness and cost‐effectiveness evaluations have revealed positive cost and societal outcomes. The model is highly compatible with the EIP approach but requires organizational support, staff induction and booster training for long‐term sustainability.

## 1 Introduction

Psychosis is associated with high disability and societal costs worldwide, contributing to over 15 million Disability Adjusted Life Years globally and impacting both productivity and health care costs (Rossler et al. 2005; The Schizophrenia Commission 2012; Lin et al. 2023; GBD 2021 Diseases and Injuries Collaborators 2024). Early intervention in psychosis (EIP) services are associated with fewer symptoms and hospital admissions, better functional outcomes and cost‐effectiveness compared to non‐EIP services (Birchwood and Fiorillo 2000; Perkins et al. 2005; Correll et al. 2018; Puntis et al. 2020; Aceituno et al. 2019). The EIP service model includes early detection, positive risk‐taking, assertive engagement, person, recovery and family focus and provision of recommended interventions (Williams et al. 2024; Bertolote and McGorry 2005). NHS England EIP guidance requires that NICE‐concordant EIP services are offered to new patients with psychosis within 2 weeks of referral, and that patients are proactively engaged to prevent them ‘falling through gaps’ between services (NHS England, the National Collaborating Centre for Mental Health and the National Institute for Health and Care Excellence 2016). However, at least 15% of patients disengage, and many more engage partially or intermittently (Robson and Greenwood 2022), with significant individual, family, health system and societal costs internationally. Key workers in EIP services are the primary clinical contact for patients and families, with a role to support and co‐ordinate care, yet there is no specific model for how key workers should engage therapeutically. A prominent ‘shared decision making’ model (Morrison et al. 2012; Tyrer 2012), is not EIP or key‐worker specific and a narrow focus on treatment decisions could be counter‐productive to EIP engagement (Greenwood, Chandler, et al. 2024). A well‐defined therapeutic engagement model is needed for EIP Key Workers, to reduce complexity and maintain consistency in service delivery.

While the EIP service model outlines what should be delivered, our Early Youth Engagement (EYE) programme defines how staff should engage young people (Greenwood, Chandler, et al. 2024; Greenwood et al. 2021; Jones et al. 2021; Greenwood, Jones, et al. 2024; Rathod et al. 2023). We have identified factors that influence engagement from the perspective of young people, EIP patients and families, including (i) communication style, (ii) social network engagement, (iii) mental health service approaches to risk, admissions and treatment, (iv) staff knowledge, attitudes and behaviours and (v) personal experiences (Greenwood, Chandler, et al. 2024; Greenwood et al. 2021). Our Delphi consultation with clinicians reached consensus on intervention components, including staff training, psychoeducational resources, family, friends and peer support protocols (Greenwood, Chandler, et al. 2024; Greenwood et al. 2021). Our feasibility‐pilot study found reduced disengagement and positive qualitative feedback with the intervention (Greenwood, Chandler, et al. 2024). The current study presents the final theoretically‐informed EYE‐2 therapeutic model for Key Workers in EIP Services. Factors affecting implementation are also presented. The model is important because in our multi‐centre, cluster randomized controlled trial, involving 1027 young people with psychosis in 20 UK EIP teams, it was associated with lower mental health care costs linked to shorter admissions, slightly lower societal costs and more days in education, training, employment and stable accommodation compared to standardized EIP alone. Societal data was in a sub‐sample and should be viewed cautiously, however, and engagement outcomes were impacted by the COVID‐19 pandemic and not affected (Greenwood et al. 2021; Jones et al. 2021; Greenwood, Jones, et al. 2024).

## 2 Method

### 2.1 Aim

The study aimed to clearly define the therapeutic ingredients, relationships between them, and proposed causal pathway to outcomes from the initial EYE Motivational–Engagement model for Key Workers in EIP services (Greenwood, Chandler, et al. 2024), and to identify factors that affect implementation. The final model then formed the basis of staff training for the EYE‐2 RCT and cost‐effectiveness study (Greenwood et al. 2021; Jones et al. 2021; Greenwood, Jones, et al. 2024).

### 2.2 Study Design

Early work to develop the model in the previous EYE project was iterative over several qualitative phases and a Delphi consultation (Greenwood, Chandler, et al. 2024). The current study presents the final cross‐sectional qualitative thematic analysis to produce the EYE‐2 model and factors supporting implementation.

### 2.3 Participant Eligibility

Participants were Sussex clinicians who attended training and took part in the original EYE pilot study over 3 years previously, purposively sampled from six geographically distinct EIP teams (Greenwood, Chandler, et al. 2024). The 3‐year gap ensured that sufficient time had passed to investigate essential components of the model and factors affecting real‐world implementation. The study aimed to recruit 12–16 clinicians to ensure diversity and representation across services and disciplines, and to ensure that common and disparate themes could be identified. All procedures contributing to this work comply with the ethical standards of the relevant national and institutional committees on human experimentation and with the Helsinki Declaration of 1975, as revised in 2013. All procedures were approved by London‐Dulwich Research Ethics Committee (Reference: 18/LO/0362 IRAS: 238744). Written informed consent was obtained from all participants.

### 2.4 Topic Guide

The topic guide (see Appendix S1) was developed by the lead author and key members of the research team (K.G., S.C., R.d.V. and C.M.), informed by Normalization Process Theory (May et al. 2022), to capture model components, implementation barriers and facilitators. The focus was on engagement as a key worker as opposed to during a therapy process.

### 2.5 Procedures

Eligible participants were identified in discussion with the EIP service manager and invited to take part. Consenting clinicians were interviewed by a research assistant (V.S.) and interviews were audio‐recorded and transcribed for analysis.

### 2.6 Epistemological and Analysis Approach

An inductive thematic analysis was applied to the transcribed data using a constructionist framework as the study was underpinned by a critical realist perspective that reality exists, but knowledge is socially constructed, with multiple differing but valid accounts of the same phenomenon (Braun and Clarke 2006; Bhaskar 2009; Hammersley 2004). The analysis followed six steps of familiarization, initial coding, searching for themes, reviewing themes, naming themes and producing the report (Braun and Clarke 2006). The thematic approach was selected as the findings go beyond experiential themes, and capture shared meaning (Braun and Clarke 2019) and the derived model was developed from multiple phases including previous research (Greenwood, Chandler, et al. 2024) and theoretical knowledge of therapeutic processes. A multiple‐coding approach captured themes from the different perspectives of core researchers (V.S., C.V., K.G., S.C., R.d.V. and C.M.), while triangulation was collaborative and aimed to facilitate reflection, while also ensuring transparency, reducing bias, confirming and reaching consensus on themes (Braun and Clarke 2019). Implementation themes were mapped onto relevant NPT constructs.

### 2.7 Researcher Stance

The research assistants who conducted the interviews and initial coding (V.S. and C.V.) were independent of the EIP service, EYE pilot and EYE‐2 trial, enabling objectivity and reduced risk of bias. The clinical EIP Research leads (S.C. and K.G.) worked in EIP services and on the EYE/EYE‐2 projects. R.d.V. and C.M. were semi‐independent and contributed qualitative (R.d.V.) and implementation science expertise (C.M.). C.M. developed the Normalization Process Theory which was used to structure implementation themes.

## 3 Results

### 3.1 Participant Demographics

Twenty‐three clinicians (six in Brighton, seven in East, 10 in West Sussex) were involved in the original EYE project between 2011 and 2015, were still in post, and were invited to take part. Eight did not respond prior to the study end, and 15 took part (three in Brighton, five in East, seven in West Sussex). Seven participants were male, eight were female, and disciplines included nursing, occupational therapy, social work, psychology and team leader roles. Specific demographic details are not provided to preserve anonymity.

### 3.2 Themes Defining the Therapeutic Model

Interviews conducted 3 years after the end of the original EYE project in Sussex revealed lasting impressions of the nature of the intervention and showed that staff still used key elements 3 years later.

Three therapeutic process themes and 10 sub‐themes summarized the key components of the EYE intervention from the Sussex clinician perspective (see Figure 1). Supporting quotes for all themes relating to the EYE model are provided in Table 1.

**FIGURE 1:**
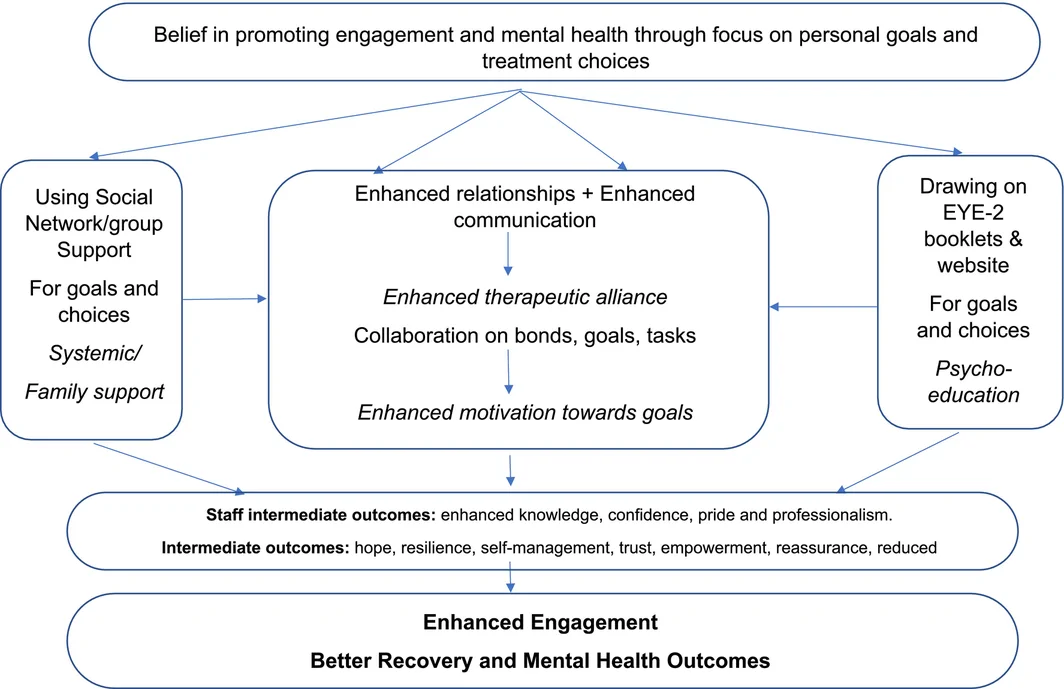
The EYE‐2 therapeutic motivational–engagement model for EIP Key Workers.

**TABLE 1:** Themes, subthemes and quotes in support of the EYE model.

Theme	Sub‐theme	Quotes
Psychoeducation	The importance of giving a physical resource [booklets]	S1: I'm trying to leave the young person with information after I'm gone so they've got a resource to look at when they're more chilled and in their own time, or with their family or friends. It's just about leaving them with something
	The value of psychoeducation	S13: One of the chaps I gave it to, the next session I had I didn't get a chance to talk about anything I'd brought with me because he had read it and digested it and he had loads of different points he wanted to raise about it—so that's how I see it you know, it's a really useful psychoeducational tool
The motivational–engagement relationship	Validation of the Key Worker therapeutic relationship as an intervention	S4: to hear from service users that the development of that therapeutic relationship with a Key Worker is so valued by them, that's hugely satisfying for us … it provided my practitioners with a lot of affirmation, that engagement is an intervention in its own right. You don't always have to be offering, a treatment to young people. There's huge therapeutic value in developing a relationship with a young person with psychosis S6: For me engagement is about a relationship with a person. Not necessarily a service initially. It may have helped keep people engaged in the service, in the treatment pathway, they felt like they had more choice, in deciding what happened to them and what treatment they wanted and had … I think, it's the relationship with the person that makes a difference
	Providing collaborative treatment choices and plans	S6: It showed that we were open to any kind of treatment that might be helpful … I think maybe what the EYE project did, was open up those conversations about alternatives to treatment … I like the fact that it wasn't just about NICE approved treatments … it was looking at other forms of treatments that maybe we wouldn't prescribe if you like or deliver ourselves S9: It's about giving people information and giving them choices and letting them make up their own mind really S2: They seemed clear and helpful [the booklets] in a way that people could understand and in kind of an empowering way, in terms of just giving people information and enabling people to get their head round sort of decision making
	Offering shared language to reduce risk	S1: It might be a prompt for someone to talk to us about something that they may not have been talking about before, particularly kind of around suicidal thoughts maybe, because that might be something that's more difficult for someone to share. They just don't want to tell you or don't know the language to tell you. And then here we go, it's all kind of written down for me. So, for a client to be able to be given a language to share that, I think, reduces risk
	A motivational listening and reflecting approach	S10: I think maybe sometimes we forget as practitioners … A lot of the service users say that having somebody who listens to them, and I think like in the training … reflecting back to [them], all of that helps with their engagement … looking at what motivates young people as well to engage with EI. We're sort of given tips I suppose about how to do that
	A respectful language and communication style	S4: Other things that were important that the EYE project identified or confirmed for us if you like. The type of language used in engagement … affording the right amount of respect for their experience, trying to frame their experience in their own language rather than superimposing a reference term that refers to illness or symptoms’
	A focus on patients' own goals	S10: Service users wanted to have like goals that followed them to the next session as well. Quite often their agenda can be set by other people, you know the parents, the education [system], criminal justice, it can feel as if for the youngsters, it might be something that is done to them rather than something they want to do. And I think the EYE stuff … helps realize you're not alone with it but also you can take ownership of it and have a positive outcome from it
An early systemic approach	Working early and assertively with families and possibly friends	S15: Often our most useful way in is through the family so we do a lot of psychoeducation and communication strategies with people like that. I think information such as in the booklets would be quite reassuring and helpful S6: We did use the family and friends' booklet. We did like a couple of sessions with the other guys in the house. I think that really supported his recovery. It was brilliant, you know. We used it in quite a structured way, I guess … with all of them. They were great. Trying to look after him and keep him safe. Trying to understand what was going on for him
Achieving expected recovery outcomes including hope, resilience and self‐management		S10: It's hopeful. It's talking about treatment and the things that we provide and being very clear about how this can really help people in their recovery S6: Some of the stuff in the EYE booklets does promote resilience for the service user. It does support them to look after themselves and not have to rely so much on [us]

#### 3.2.1 Psychoeducation

Two themes related to the value of psychoeducation and giving a physical resource.

- *The importance of giving a physical resource* [booklets]

Clinicians prioritized the act of giving a physical resource, something tangible, that could be reviewed in the patient's own time, and that left a positive lasting impression for a person in need, over sharing the same resource via the website.

ii *The value of psychoeducation*

They also valued the accessible and evidence‐based psychoeducational content of the resource that was co‐produced with clinicians, families and service users and enabled service users and families to meaningfully shape their engagement. The resources include myth‐busting booklets on (i) Mental Health and Help‐Seeking, (ii) EIP Services and (iii) Treatment Choices for Unusual and Distressing Experiences and Psychosis (140 pages) for patients and the (iv) Friends and Family booklet.

#### 3.2.2 The Motivational–Engagement Relationship

Six themes related to the enhanced therapeutic relationship, communication and focus on patients' own goals.

iii *Validation of the Key Worker therapeutic relationship as an intervention*

The intervention incorporated a central role for the key worker therapeutic motivational–engagement relationship with the patient, focussed on the patient's own positive treatment goals. This foregrounding of the keyworker relationship was validating and elevated the importance of therapeutic engagement as an intervention in its own right, alongside standard treatments.

iv *Providing collaborative treatment choices and plans*

A key component of the motivational–engagement relationship was the provision of co‐produced evidence‐based treatment choices, including medication options informed by side effect profiles, standard psychological and vocational treatments, and alternatives such as peer support, self‐management and spiritual faith‐based approaches. Key workers were enabled to provide a menu of options to support treatment decision‐making. Interventions could be selected collaboratively in line with the patient's goals. Reviews could consider treatment offers and progress. The honest information and quotes in the resources facilitated trust, hope and empowerment for patients and families.

v *Offering shared language to reduce risk*

The lasting impression was that the EYE intervention offered a shared language to reduce risk. The intervention was critically framed and training provided to support conversations about psychosis experiences, goals and treatments in the context of risk. Concerns and threats to engagement and risk identified by patients and their families, such as differing perspectives on hospitalization and medication, were proactively discussed in the resources and cultural preferences subsequently incorporated to further enhance engagement.

vi *A motivational listening and reflecting approach*

Key workers developed a greater understanding of the processes of motivational engagement, including how to listen actively to a patient's motivations for engagement and their goals for personal recovery and how to encourage curiosity and thought about more complex issues that could hold the patient back.

vii *A respectful language and communication style*

Alongside and inherent in the motivational engagement approach was the recognition that respectful language is critical, and that engagement can be enhanced by framing experiences, motivations and goals in the patient's own positive language.

viii *A focus on patients' own goals*

A final core focus was the identification of therapeutic goals that were important to the patient and that influenced their engagement, as opposed to those arising solely from parents, school, college, employer or the criminal justice system.

#### 3.2.3 An Early Systemic Approach

The ninth theme related to the systemic approach.

ix *Working early and assertively with families and possibly friends*.

Critical to the EYE systemic approach was the early incorporation of family, friends, peers, social group members or others in the person's social network who could support the patient with their particular treatment or recovery goals.

#### 3.2.4 Achieving Meaningful Recovery Outcomes

The final theme in the model relates to expected recovery outcomes.

x *Achieving expected recovery outcomes including hope, resilience and self‐management*.

Finally, there was a common theme relating to the way in which the EYE intervention could facilitate recovery outcomes including hope, resilience and self‐management.

### 3.3 Facilitators and Barriers to Implementation of the Therapeutic Model

Eleven themes reflected facilitators and three reflected barriers to implementation. These were mapped onto the Normalization Process Theory (NPT) framework (see Table 2 for an outline of implementation themes and quotes and Table 3 for the mapping of themes onto the NPT framework). While clinicians defined three core components of the model, their reflections on implementation revolved largely but not exclusively around the most tangible psychoeducation component.

**TABLE 2:** Themes and quotes in support of implementation barriers and facilitators.

NPT construct	Theme	Quotes
Coherence	Value of the EYE model	I just think it helps us as a service come over more sort of professional and, I think people like it. I think it just adds maybe a bit more value to us. I think it's useful as well, where I think, particularly when you might be getting close to a stage where you feel a bit stuck with someone. Erm, that maybe, at that stage it's er, maybe that would be where you would look at it more and say look it helps you broaden your perspective. It's got all of the information somebody needs in one place, and it's easily accessible, and it's not written by us, which people quite like.
	Positive emotional impact for clinicians	That attitude of excitement and hope definitely affected our use of them initially … I guess for me the thing that sort of stood out … that sense of hope and excitement. I guess it was the excitement of having something which was so hopeful and inclusive.
	Importance of service user involvement in development	It's about being inclusive, collaborative, not using jargon, providing as much relevant information as we can for people at the right time, at the right pitch, the right tempo. To have that expressed in a language that's been built and put together by young people, er, experiencing psychosis, that's immensely useful. I was very pleased to see a very comprehensive consultation with young people around all that information. So it very much felt like in the design of the booklets, the style of the information, the presentation, the website development, you know, it all seemed to be driven by young people themselves.
	Compatibility with the EIP approach	The EYE project is a perfect fit for our style of working, our ethos, our approach, our values. The goals are the same: remove barriers to engagement, encourage positive meaningful engagement, encourage collaboration, bust stigma and myths around mental health problems.
Cognitive participation and adoption	Regular use of the resources	We like using them [resources]. We use them regularly.
	Informal handovers to new staff and patients	When new staff are inducted, these are the contents of the assessment pack … the EYE project leaflets are part of it. My expectation would be that new members of staff take the time to read them.
Collective action and wider support	Staff confidence linked to the resources	I think it's helped boost confidence in the way that we approach the work.
	Systematic ways of using the resources	It was a routine part of our assessment pack. We would hand it out at that particular point, routinely … or maybe send it out with a first care plan. It's not just about what we offer, it's about what we maybe have already offered, and people have turned down. It could just be a gentle prompt to allow families to know we've tried. If a parent or a service user has contacted me with questions about different medications and what other things they can try, I'll often send them the Treatment Choices booklet, and a reference to certain pages that I think are going to be helpful. The Treatment Choices booklet is a very important part of shaping the early care plan, and really inviting young people and their families to be collaborative in putting a care plan together. Then you get onto the next level which is a little bit more in detail about what, let's do a bit of psycho‐ed around what psychosis is, let's think about what maybe some of the treatment options might be … then think about maybe you know, erm, recovery plans, you know with people and think about your own resilience, and kinda moving on to, you know, a place where you don't really need us anymore.
	Different staff engaging in different ways	We all work differently but we all work equally hard in engagement.
	Organizational support (and the need for booster training, reminders, updates and induction)	The service manager and the clinical lead … They all supported it and made it happen. Leaders in the service having awareness and having it as part of their general agenda, that there's a resource, don't forget about it, it has its use for X, Y, Z. We need prompts don't we. We're busy, we've got a lot of things to juggle
Reflexive monitoring and appraisal	Positive staff, carer and patient feedback	We've got feedback from young people and their families that [the booklets] they're helpful. Subjectively I think we're, we're doing better, we're losing less people through drop out.
Barriers	Patient characteristics and processes	Sometimes we have clients with a fairly fragile engagement, with quite poor insight who almost take exception to the idea that they have a mental illness. We have to tread very carefully and be very subtle and discreet in those circumstances.
	Staff crises and turnover	My sort of experience is that the more firefighting people are doing, if they're practitioners, then the less things are gonna be used, you know like the materials for instance would be used less if people were going from crisis to crisis. I don't always feel as though I have the time to keep on reinforcing it because the turnover has been quite high in my team and it's just, it's another thing to keep on explaining.
	Resource characteristics	If somebody's concentration was so poor, then written information, sometimes, they can't handle it.

**TABLE 3:** Mapping the EYE model onto NPT as a determinant framework.

Implementation mechanisms: what staff will need to do to implement the EYE model	Implementation outcome constructs: how things can change				Sources of potential success for the EYE model and resources
	Intervention performance: what practices will change as the result of EYE model and its components being operationalized, enacted, reproduced, over time and across settings?	Relational restructuring: how could working with EYE model and its components change the ways people are organized and relate to each other?	Normative restructuring: how will working with EYE model and its components change the norms, rules and resources that govern practice?	Potential for sustainment/normalization: how will EYE model and its components become routinely incorporated in practice?	
Coherence: how people work together in everyday settings to understand and plan implementing the EYE model	(+) Compatibility: the EYE Model was seen as compatible with a good EIP approach, provided detail regarding how to engage people and endorsed and offered a succinct summary of the service	(+) Positive emotional impact: Clinicians described feeling happiness, pride, hope, excitement, vibrancy and motivation	(+) Value of the model: clinicians valued the psychoeducational resources which were trusted, evidence‐based and user‐friendly	(+) Value of the model: staff liked that information was ‘at their fingertips’ and valued the ‘act of giving’ booklets (−) Staff crises and turnover: Staff were less likely to use the model if they themselves were stressed, ‘fire‐fighting’ or managing crises	The EYE model and resources were highly trusted and appeared to be minimally disruptive to both practitioner identity and practice workflow
Cognitive participation: how people work together to create networks of participation and communities of practice around the EYE model		(+) Informal handovers: new staff were inducted into the model and provided with the psychoeducational resources (−) Patient characteristics: patient mental state and motivation to participate in the EYE Model of care	(+) Staff confidence and positive feedback: legitimisation of the approach occurred through staff confidence, feedback and continued use		The EYE Model was seen as an appropriate and legitimate mode of practice.
Collective action: how people work together to enact the EYE model and its components	(+) Regular and systematic use: EYE resources were used as an introduction, a starting point, at assessment, with the first care plan, as a reminder, to reinforce key messages, in reviews, as a checklist for what had been offered, to educate other professionals, in a structured way with friends and flexibly dependent on illness phase	(+) Different staff engage in different ways: Key workers engaged holistically, but also used resources relevant to their individual discipline	(+) Organizational support: clinicians noted the critical importance of strong clinical and managerial leadership within teams, supported by booster training, updates and induction for new staff in order to maintain the model	(+) Regular and systematic use: Reading the resources boosted confidence (−) Resource characteristics: lack of confidence in technology impacted on website use	EYE Resources appeared to possess a high degree of interactional and skill‐set workability—giving robust and trusted structure to practitioner–client interactions
Reflexive monitoring: how people work together to appraise the EYE model and its components				(+)Staff confidence and positive feedback: Clinicians were confident that the EYE model worked	Clinicians were confident that the EYE model worked

*Note:* Shaded cells denote headings and conclusions. Unshaded cells present study findings.

#### 3.3.1 Potential Facilitators to Implementation

##### 3.3.1.1 Coherence With EIP Practice

Four themes related to the coherence of the model with current EIP practice.

- *Value of the EYE model*

Clinicians valued the psychoeducational resources which were trusted, evidence‐based, user‐friendly, professional, comprehensive, holistic and promoted understanding in patients, families and new clinicians. Staff liked that information was at their fingertips and, as described previously, valued the act of giving booklets.

ii *Positive emotional impact for clinicians*

Clinicians described feeling happiness, pride, hope, excitement, vibrancy and motivation, and felt that patients and carers were reassured, comforted, contained, empowered and less worried as a result of receiving the psychoeducational resources.

iii *Importance of service user involvement in development*

Service user involvement and the impact on language and design helped clinicians to connect with new patients who in turn valued the service user experiences and quotes.

iv *Compatibility with the EIP approach*

The EYE model was seen as compatible with a good EIP approach in terms of ethos, values and goals, while providing detail regarding how to engage people.

##### 3.3.1.2 Cognitive Participation and Adoption

Two themes related to Cognitive Participation and adoption of the model as a legitimate Key Worker role: *(v) Regular use of the resources and (vi) Informal handovers to new staff and patients*.

Legitimisation of the approach occurred through staff confidence, feedback and regular continued use. New staff were inducted into the model and provided with the psychoeducational resources.

##### 3.3.1.3 Collective Action and Wider Support

Four themes related to Collective Action, wider buy‐in and management support.

vii *Staff confidence linked to the resources*

Reading the resources boosted confidence, although conversely not reading them meant staff were less likely to use them. Lack of confidence in technology impacted on website use.

viii *Systematic ways of using the resources*

EYE resources were used as an introduction, a starting point, at assessment, with the first care plan, as a reminder, to reinforce key messages, in reviews, as a checklist for what had been offered, to educate other professionals, in a structured way with friends, and flexibly dependent on illness phase.

ix *Different staff engaging in different ways*

Key Workers engaged holistically, but also used resources relevant to their individual discipline; for example, a nurse focused on physical health and a support worker on activities.

x *Critical organizational support*

The critical importance of strong clinical and managerial leadership was noted, and the need for booster training, reminders, updates and induction for new staff in order to maintain the model.

##### 3.3.1.4 Reflexive Monitoring and Appraisal

One theme related to Reflexive Monitoring or appraisal at the individual level: (xi) *Positive staff, carer and patient feedback*.

Feedback from young people and families that the approach and resources were helpful meant that clinicians were more confident that it worked.

#### 3.3.2 Potential Barriers to Implementation

Finally, three themes related to barriers to implementation: *(xii) Patient characteristics and processes; (xiii) Staff crises and turnover* and (xiv) *Resource characteristics*.

Specifically, a patient's mental state, risk and motivation impacted use of psychoeducation resources, while concerns about stigma and confidentiality were barriers to engaging families and friends. Staff were less likely to use the model if they were stressed, ‘fire‐fighting’ or managing crises, had insufficient time to engage, or if staff turnover was high, leading to staff shortages. Finally, sometimes the booklets were seen as too much written information in one go.

The core therapeutic processes and factors affecting implementation were the primary focus of the EYE‐2 staff training and intervention manual. Adaptations to support implementation included the provision of posters, memory prompts, supervision checklists, 6‐monthly booster training, content summaries for booklets, with guides for how and when to use them, and individual downloadable handouts for each treatment option from the treatment choices booklet. Peer‐led social groups were developed in collaboration with the McPin Foundation and are outlined in a separate Social Groups Booklet.

## 4 Discussion

The final model clearly defines three core therapeutic processes intended to promote engagement and recovery through (1) an enhanced central therapeutic alliance, with increased focus on the therapeutic relationship, collaborative communication, choice in the context of risk, and motivation to support the young person with their own goals. This strengthened alliance and focus on personal goals are supported by (2) psychoeducation approaches, utilizing the EYE‐2 resources, website and booklets systematically (see www.likemind.nhs.uk, accessed 13/10/2026) to identify choices and desired recovery outcomes and (3) systemic social network approaches which can be harnessed to further support the patient to identify and reach their individual recovery goals. The role of the enhanced therapeutic relationship is clearly central, incorporating communication, alliance and motivation‐building. Greater emphasis is placed on person‐centred psychoeducation as a process of change, and with some emphasis also on harnessing the broader supportive social network. The model informed the subsequent EYE‐2 staff training and intervention manual (Greenwood, Jones, et al. 2024).

Implementation was facilitated by model Coherence in terms of perceived value and positive emotional impact for clinicians, service user involvement in model development and compatibility with the standard EIP approach. Implementation was further supported by Cognitive Participation, which involved regular use of the approaches and informal handovers to new staff. Collective action was in evidence in the development of systematic processes for resource use, as well as staff engagement, confidence and critical organizational support, and Reflexive Monitoring occurred through appraisal of positive staff, patient and carer feedback. Barriers to use of the model included specific patient characteristics, staff crises and turnover, and resource characteristics. Actions to support implementation were incorporated into the EYE‐2 staff training and manual (Greenwood, Jones, et al. 2024).

The EYE‐2 Key Worker therapeutic model is consistent with but extends the psycho‐social developments in the psychosis literature. Treatment choice, collaboration and shared decision making are increasingly central to engagement, although care should be taken to avoid a narrow focus on medication only, as a broader focus on goals and holistic approaches is important to young people. Family‐oriented open dialogue approaches in first‐episode psychosis have a growing evidence base with potential for positive long‐term outcomes (Bergström et al. 2018). The preferential knowledge and unique role afforded to siblings and the added value of involving friends of young people in their treatment have been emphasized (Harrop et al. 2015; Bowman et al. 2017). There are early indications of value in incorporating peer workers in EIP services (Robinson et al. 2010; Nguyen et al. 2021). Work by our group has demonstrated the value of hopefulness in therapeutic relationships and social inclusion (Berry and Greenwood 2015). Finally, psychoeducation is recognized in psychosis and EIP services as an intervention in its own right, with impacts on engagement (adherence) with treatment approaches, relapse, social and global outcomes (McGorry 1995; Xia et al. 2011).

This study is important in defining for the first time the key components of a therapeutic Motivational–Engagement Model for Key Workers in EIP services. The model is timely, especially in the UK where recent events have highlighted the importance of engagement with service users and families, including (1) care delivery in the context of disengagement; (2) co‐produced care‐planning; (3) family engagement; (4) access to peer support; (5) understanding risk and (6) implementation of recommendations (NHS England 2025). The EYE model, training and psychoeducational resources are well‐placed to support such recommendations. The EYE model has a clear focus on care delivery in the context of disengagement, ensuring that intervention approaches are co‐produced with people with lived experience. The model provides resources to enable service users and their families to contribute to care plans. It provides a language for service users and resources for services to aid collaboration in the context of risk. It provides evidence to support positive family engagement and robust peer support. The EYE implementation framework has potential to facilitate implementation.

### 4.1 Strengths and Limitations

The study recruited clinicians who were still in post several years after the end of the previous EYE project, which enabled us to capture core model components and factors relevant to long‐term sustainability and implementation. This model is the first to clearly articulate the therapeutic role of the Key Worker in engaging patients with psychosis in an EIP service. Our subsequent RCT and cost‐effectiveness study has revealed that even with modest implementation, this model may increase time spent in education and training by 6 weeks, and time spent in paid employment by 1.5 weeks per year (Greenwood, Jones, et al. 2024). The indicated societal outcomes should be viewed cautiously due to smaller sample sizes but may hold potential given that the disease burden associated with psychosis has remained unchanged over 40 years (GBD 2019 Mental Disorders Collaborators 2022). Therapeutic communication in the context of risk may reduce time spent in inpatient settings for those who are admitted, and reduce costs for unplanned care (Greenwood, Jones, et al. 2024). However, time elapsed since the original EYE training meant that some people had not remembered all elements of the model, and those who moved out of EIP services or did not participate may have held different perspectives on the model. Components of the EYE intervention have been identified as important by service users, carers and clinicians, and reported qualitatively and in our pilot study to facilitate engagement. There was no effect on engagement in our main trial, but implementation was substantially impacted by the COVID‐19 pandemic. Effective implementation is critical and can be facilitated, in routine contexts, using the approaches described. The current study comprised a comparatively small sample which could be seen as a limitation. However, the model was in fact developed from an iterative process, building on three phases of the initial EYE project, involving 22 young people, 29 patients, 31 carers and 107 clinicians in total (Greenwood, Chandler, et al. 2024), in addition to the 15 clinicians in the current 3‐year follow‐up study.

### 4.2 Summary

This study defines, for the first time, the key components of a Therapeutic Motivational–Engagement Model for Key Workers in EIP services. The clarity regarding core therapeutic processes required to support young people in treatment has the potential to enhance quality and reduce complexity in the Key Worker role. This clarity is also important because our work has shown that role confidence and security impact on therapeutic relationships in psychosis (Berry and Greenwood 2016), which in turn impact outcomes. The model can be used to facilitate the induction of new staff and maintain consistency over time, and is well‐liked and easy to implement. It is supported by a training programme and psychoeducational resources (www.likemind.nhs.uk, accessed 13/10/2026) adapted for people from minority ethnic, spiritual and cultural backgrounds and for people whose first language is not English (Rathod et al. 2023). However, implementation may be harder as patient risk and staff stress increase, and long‐term sustainable implementation requires organizational support and regular induction and booster training.

## Funding

This work was supported by the National Institute for Health and Care Research Health Services and Delivery Research programme awarded to K.G. (Reference: 16/31/87).

## Disclosure

The lead author and manuscript guarantor (K.G.) affirms that the manuscript is an honest, accurate and transparent account of the study being reported; no important aspects of the study have been omitted and that any discrepancies from the study as planned have been explained.

## Conflicts of Interest

K.G. has received NIHR funding for previous research, and has received funding from the University of Sussex, Sussex Partnership Foundation Trust and South‐East Network for Social Sciences (SeNSS) for work linked to this project. She has received funding for a conference presentation from Boehringer Ingelheim. S.R. has received consultancy fees and honoraria from various industry providers, does medicolegal work and sits on an industry advisory board. P.P. has received NIHR funding, industry funding, book loyalties and honoraria from universities for lectures and presentations. P.F. has received previous NIHR research funding and is clinical advisor to the National Clinical Audit of Psychosis. He has sat on HTA Prioritization and funding panels. P.G. has received funding from NIHR and Wellcome and is a member of an expert international advisory committee. D.F. has received previous NIHR research funding. E.P. has received funding from the Medical Research Council and NIHR. L.J. has received funding from NIHR for research, received book royalties and payment for workshops. S.B. has received previous NIHR funding. C.M. has received financial reimbursement for travel to present on NPT. K.G., S.C., S.N., L.J. and A.O'D. either currently or previously worked in early intervention in psychosis services, and B.L. and P.F. have been regional EIP leads. The other authors declare no conflicts of interest.

## Supporting information


**Appendix S1:** The interview topic guide.

## Data Availability

Fully anonymized data will be available from K.G. upon reasonable request, subject to submission and approval of a research proposal and review and contract with Sussex Partnership NHS Foundation Trust.
