# Supplementary material for: The Early Youth Engagement (EYE‐2) Intervention in First Episode Psychosis Services: Definition and Implementation of a Therapeutic Motivational–Engagement Model for Early Intervention in Psychosis Key Workers
Source: Early Interv Psychiatry. 2026 Oct 16;20(10):e70270. doi: 10.1111/eip.70270 (PMC13674719; doi:10.1111/eip.70270)
Supplement: Supplementary file 1 [file EIP-20-0-s001.docx]

**Appendix 1 The Interview Topic Guide**

**Outline topic guide**

**Introduction:** The Early Youth Engagement in First Episode Psychosis Study is a study that we ran in Sussex EIP services between 2011-2015. It focussed on improving the engagement of young people in EIP services. EIP services here were involved in focus groups, and interviews to develop the intervention approach. It included a training day, resources and booklets.

**Topic Guide:**

1. **Informational Resources** - What do you know or remember about the original EYE project training, intervention approach and resources?
   a. What are the main engagement approaches?
   b. Is there anything else you can remember?
   c. What are the main resources?
   d. Is there anything else you can remember?
2. Are you currently using anything from the original EYE project? If so what?
   a. in terms of approaches/ideas that you learnt from the project and training?
   b. in terms of EYE resources?
3. **Material Resources** - How does availability of the resources (both actual resources like booklets, computers with wifi, as well as your time) impact on whether you use the EYE approach or not?
4. **Workability and Integration (institutional)** - How do the approaches and materials from the EYE project fit with the way that you feel you ought to be working, and perhaps with the ways that you feel your peers think you ought to be working?
   a. What gets in the way of using the approaches/materials?
   b. What is it that encourages you to use the approaches/materials?
5. **Social Norms (institutional)** – How does using the EYE approach and materials change the way that you work/the overt or covert rules about how you work?
6. **Social Norms (institutional)** How does the Consistency/inconsistency of the EYE approach fit with risk and safety issues
7. **Social Norms (institutional)** How is use of the EYE approach/resources affected by other service demands/pressures? (caseloads?)
8. **Social Norms (social network)** Who uses the approach and materials? What governs who uses the approaches and materials? Who works hardest at engaging young people in the service?
9. **Social norms (social network)** Thinking about clinicians in the team, how do the attitudes and behaviours of the staff, service users or carers around them towards the EYE approach and resources influence whether they use it or not? Might anyone strongly influence their views recently or in the past?
10. **Individual Intentions** Thinking about clinicians in the team, how consistent are the EYE project approach/resources with their beliefs, attitudes and behaviours?
11. **Shared intentions** Thinking about clinicians in the team, how consistent are the EYE project approach/resources with the beliefs, attitudes, behaviours, priorities of the team/service as a whole?
12. **Workability** What does using the EYE approach and resources involve doing? How possible is it to use these approaches? How do space/staff/caseload issues impact?
    a. Use of website
    b. Use of booklets – help-seeking, EIP, friend and family, treatment choices?
    c. Use of motivational approaches focused on needs/goals?
    d. Use of open, honest communication in the context of difficult issues? (hospital/medication)
    e. Offering treatment choices?
    f. Involvement of the participants whole social network including key family and friends?
    g. Use of peer workers/running of social groups?
    h. Use of schools pack?
13. **Integration** – How does use of the EYE approach and resources fit with other demands on your time, and other things that you need to do when working with service users in EIP?
14. **Coherence** – What has helped you to know how to use the EYE approach and resources? Is there anything else that could be provided to help you to know how to use the EYE approaches/resources?
15. **Cognitive participation** – What do you do that helps you to put the EYE project approaches/resources into practice? What do you do to help others to use the approaches and resources? Have you introduced any new staff to the approach and resources? What have you told them?
16. **Cognitive participation** – Is there anything that you do that gets in the way of or stops you using the EYE approaches and resources? **Timing** – are there times when you wouldn’t use the EYE approaches and resources?
17. **Collective Action** – What have you done to help you to become better able to use the EYE approaches and resources? Or to engage better with young people using the service?
18. **Reflexive monitoring** – **How do you know whether the approaches and resources that you use that are part of the EYE project are helpful?** How do other staff in the service know whether these approached are helpful or not? How do you learn from this and apply it when working with more young people and their families in the future?
19. Is there anything else that we should include in the training and manual for new staff delivering the EYE project to help ensure that staff can make the best use of this approach in the future?
20. Is there anything else you wanted to add that we haven’t already covered?

**Ending –** thank the person for their time, explain again what will happen to their information, how it will be used and the value of this study. Ask the person whether they would like copies of the materials when completed and whether they would like to remain in touch and receive updates about the study. Provide a study leaflet for them to contact us if they need to at any future time.
